# Evaluation of paediatric nursing-sensitive outcomes in an Australian population using linked administrative hospital data

**DOI:** 10.1186/1472-6963-13-396

**Published:** 2013-10-08

**Authors:** Sally Wilson, Alexandra P Bremner, Yvonne Hauck, Judith Finn

**Affiliations:** 1 School of Population Health, The University of Western Australia, 35 Stirling Hwy, Perth 6009, Western Australia; 2 School of Nursing and Midwifery, Curtin University, GPO Box U1987, Perth 6845, Western Australia; 3 Prehospital, Resuscitation and Emergency Care Research Unit, Faculty of Health Sciences, Curtin University, GPO Box U1987, Perth 6845, Western Australia; 4 Department of Epidemiology and Preventive Medicine, Monash University, Alfred Centre, 99 Commercial Road, Melbourne, VIC 3004, Australia

## Abstract

**Background:**

Research into nursing-sensitive outcomes using administrative health data has focussed on hospitalised adults. However, we developed algorithms for the identification of 13 paediatric nursing-sensitive outcomes, which we seek to examine for clinical utility. The aims were to determine the rates of paediatric nursing-sensitive outcomes in a Western Australian hospital and ascertain sociodemographic and clinical characteristics associated with a greater risk of developing nursing-sensitive outcomes in hospitalised children.

**Method:**

A retrospective cohort study used linked administrative data of all Western Australian children ≤18 years admitted to the only tertiary paediatric hospital in Perth between 1999 and 2009. Rates per 1,000 hospital separations and per 10,000 patient days were calculated for the following nursing-sensitive outcomes: lower respiratory tract infection (LRTI), gastrointestinal (GI) infection, pneumonia, sepsis, arrest/shock/respiratory failure, central nervous system complication, central venous line infection, infectious disease, pressure ulcer, failure to rescue, surgical wound infection, physiologic/metabolic derangement, and postoperative cardiopulmonary complications. Poisson multiple regression models were fitted to estimate rate ratios (RR) and 95% confidence intervals (CI) for suspected risk factors.

**Results:**

Linked records of 129,719 hospital separations were analysed. Rates ranged from 0.5/1,000 for pressure ulcer to 14.0/1,000 hospital separations for GI infections. Age was significantly associated with the risk of a nursing-sensitive outcome: compared with adolescents, toddlers had greater risk of GI infection (RR 9.89; 95% CI 6.24, 15.69); infants had 7.74 times greater risk of LRTI (95% CI 5.11, 11.75), while neonates had lower risks for sepsis (RR 0.26; 95% CI 0.08, 0.90) and physiologic/metabolic derangement (RR 0.12; 95% CI 0.04, 0.35). The risk of surgical wound infection was 7.78 times greater (95% CI 5.10, 11.86) for emergency admissions than elective admissions.

**Conclusions:**

Seven of the 13 defined nursing-sensitive outcomes occurred with sufficient frequency (>100 events over the 10 year study period) to be potentially useful for monitoring the quality of nursing care. These nursing-sensitive outcomes are: LRTI, GI infection, pneumonia, surgical wound infection, physiologic/metabolic derangement, sepsis and postoperative cardiopulmonary complications. When used for quality improvement or to benchmark with other agencies, data need to be adjusted for, or stratified by age and admission type, to ensure equitable comparisons.

## Background

There is growing interest in Australia in developing clinical indicators to measure the quality of health care [1,2]. As nurses make up more than 50% of the Australian health workforce, [3] it is not surprising that so-called 'nursing-sensitive indicators’ have been proposed. These indicators measure the structures, processes and outcomes of nursing care, [4] and the latter of which are defined as “changes in health status upon which nursing care has had a direct influence” [5,6]. Algorithms for detecting nursing-sensitive outcomes in administrative hospital records, have been developed, [7,8] and used to measure the quality of nursing care in North America [9-11], Europe, [12] New Zealand [13] and Australia [14-16].

Most of the work in nursing-sensitive outcomes has involved adult patients. Some researchers have included paediatric patients within predominantly adult populations when measuring the quality of nursing care [17-19], however these nursing-sensitive outcomes have only been validated in adult acute care [7,8], so may not be applicable in the paediatric context. Although paediatric nursing-sensitive indicators [4,20] and generic paediatric outcome indicators have been developed, our previous paper and was one of the first to report algorithms for defining paediatric nursing-sensitive outcomes using linked administrative hospital data [21].

Hospital discharge summaries are useful for research in this field as they commonly include demographic information about the patients as well as standardised coding of their principal diagnosis, comorbid diagnoses, procedures performed and complications that arise during hospitalisation [22]. However, there are many specific challenges in identifying nursing-sensitive outcomes in paediatrics. Children are generally less likely to be admitted to hospital: they are a healthier group with fewer chronic illnesses, and have diverse physiological, psychosocial and developmental stages [23]. Furthermore, unlike the Charlson or Elixhauser Comorbidity scores [24,25] for adults, there is no established paediatric comorbidity classification system.

There are also generic challenges related to the use of administrative hospital records when determining nursing-sensitive outcomes. Most importantly is the challenge of differentiating outcomes due to the patient’s premorbid or comorbid state, or other social or environmental factors, rather than nursing care. This challenge has been addressed through the development of complex algorithms that exclude an adverse patient outcome from being considered a nursing-sensitive outcome in the presence of certain comorbid conditions or patient characteristics. These algorithms are usually applied to a single hospital record, so accuracy of nursing-sensitive identification is contingent on the comorbid condition also being coded on that record. However, in our previous work using linked data [21], we found that recording of comorbid conditions was not consistent. For example, we found that 43% of children with pressure ulcers had a form of paralysis recorded on a previous hospital admission, but not on the hospital admission which identified the supposed nursing-sensitive outcome of pressure ulcer [21]. Without the ability to 'look back’ at prior hospitalisation records (using linked data), we could have incorrectly identified these adverse events as nursing-sensitive and thus over-estimated the rate of nursing-sensitive pressure ulcers. Building on previous work with nursing-sensitive outcomes in adult patients, [8] generic paediatric quality outcomes [26,27], and incorporating the benefits of linked hospital data, we have proposed 13 possible paediatric nursing-sensitive outcomes and their corresponding inclusion and exclusion algorithms for use with linked administrative hospital data (Table 1).

**Table 1:** Definitions of paediatric nursing-sensitive outcomes

**Nursing-sensitive outcome**	**Numerator**^**a**^	**Denominator**	**Key exclusions**
Lower respiratory tract infection (LRTI)	Lower respiratory tract infections, bronchiolitis (ICD^b^ B97.0, B97.4, B97.89, > = J20.0 and < =J20.9, J21.0, J21.8, J21.9, J22., J85.1, J85.2, J86.0, J86.9)	All medical and surgical inpatients	MDC4-respiratory conditions
		LOS > =3 days	All pneumonias
Gastrointestinal (GI) infection	Rota virus and other GI infections (ICD A02.2, > = A03.0 and < = A03.9, > = A04.0 and < = A04.9, A08.0, A08.1, A08.2, A08.3, A08.4, A09., A09.9)	All medical and surgical inpatients	Principal diagnoses of volume depletion, disorders of fluid and electrolytes or acid base
		LOS > =5 days	
			(ICD E86., E87. > = E87.0 and < =E87.8)
			MDC6-GI system
			MDC18-infectious and parasitic
Pneumonia	Aspiration, post-operative, hypostatic, bacterial, broncho and unspecified pneumonias: (ICD > =J14. and < =J15.9, J18.0, J18.1, J18.2, J18.8, J18.9, J69.0, J69.8, J95.8, J95.9)	All medical and surgical inpatients	MDC4-respiratory conditions
			All diagnoses of probable community acquired pneumonia (ICD > =J10. and < =J10.8, > = J11. and < =J11.8, =J12. and < =J12.9, J13., J15.7, J16.8, > = J17. and < =J17.8)
			Aspiration pneumonia and epilepsy (ICD J69.0, 69.8, > = G40. and < =G40.9, > = G41. and < =G41.9)
			All diagnoses of epilepsy found in 'look back’ period
Surgical wound infection	Surgical wounds, including surgery post traumatic injury plus those found in 30 day 'look forward period’ (ICD T79.3, T81.4, T81.41, T81.42)	All surgical inpatients	
		Total discharges only	
Physiologic/metabolic derangement	Diabetic hyperosmolarity/acidosis: (ICD > E10.1, E 10.11, E11.1, E11.11, E15.)	All surgical inpatients	If principal diagnoses diabetes: (ICD > =E10 and < =E14, E15.)
			Principal diagnosis of trauma and burns: (ICD > =S00. and < =T32.9)
	Disorders of fluid, electrolytes, acid–base: (ICD E86., > = E87. and < =E87.8)		
			MDC5 circulatory system, MDC7 hepatobiliary and pancreas, MDC10 endocrine and metabolic disorders, MDC11 kidney and urinary tract
	Shock from a procedure: (ICD T81.1)		
	Anuria/oliguria: (ICD R34.)		
			No procedure code
			If principal diagnosis cardiac arrhythmia/arrest (ICD > =I46. And < I50.) or GI haemorrhage (ICD K92., K92.1, K92.2)
Sepsis	Septicaemia; bacteraemia (ICD > =A40. and < A42., A49.9, R78.81)	All medical and surgical inpatients	MDC17-cancer and all diagnosis of cancer in 2 year look back period
		LOS > =3 days	
			Infection related admissions (DRG 2.0-4.2: B72Z, D63A, D63B, D64Z, D66A, D66B, E62A, E62B, E62C, F61Z, G07A, G07B, I64A, I64B, I67A, I67B, J11Z, J64B, L40Z, L41Z, L63C, M62A, M62B, N61Z, S63A, S63B, S64A, S64B, T01A, T01B, T01C, T60A, T60B, T61A, T61B, T64A, T64B)
	Look back period for cancer		
			(DRG 5.0-6.0: B72A, B72B, D63A, D63B, D64Z, D66A, D66B, E62A, E62B, E62C, F61Z, G07A, G07B, I64A, I64B, I67A, I67B, J11Z, J64B, L40Z, L41Z, L63A, L63B, L63C, M62A, M62B, N61Z, S64B, S65A, S65B, S65C, T01A, T01B, T01C, T60A, T60B, T61A, T61B, T64A, T64B)
Postoperative cardiopulmonary complications	(ICD > =I26. and < = I26.99, I44.2, I46.0, I46.1, I46.9, > = I50. and < =I50.9, > = I97. and < =I97.9, J80., J81., > = J95. and < =J95.4, J95.8, > = J96. and < =J96.9, > = J98.0 and < =J98.3, R09.2, > = T80.0 and < =T80.2, T81.7, T81.72, T82.8, T82.9)	All surgical inpatients	MDC3-Ear, nose, mouth and throat conditions
			MDC4-respiratory conditions
			MDC5-circulatory conditions
Arrest/shock/respiratory failure	Arrest (respiratory, cardiac), respiratory failure, shock (ICD I46.0, I46.1, I46.9, J18.2, J80., J81., J95.1, J95.2, J96.0, J96.9, R09.2, > = R57.0 and < R58)	All medical and surgical inpatients	MDC4-respiratory conditions
			MDC5-circulatory conditions
	Procedures (ACHI 92042–00, 92052–00, 92053–00)		
Central nervous system (CNS) complications	Coma and stupor; acute delirium; reactive confusion; reactive depression (ICD F05.0, F05.8, F05.9, F43.2, F43.9, F44.88, F51., R40.0, R40.1, R40.2, R40.4, R41.0, R41.8, R45.1, R45.4,)	All medical and surgical inpatients	MDC1-neurological
			MDC19-mental illness
		Age >6 months	
			MDC20-substance abuse
			MDC21-injuries, poisoning and toxic effects of drugs
			Any procedure of sleep study (ACHI 12203.00)
Central venous line (CVL) infection	Infection due to central venous catheter as per AHRQ (ICD > =T80.2 and < =T80.29, > = T82.7 and < = T82.79)	All medical and surgical inpatients	MDC17-cancer
			Weight < 500gm
		LOS > =2 days	
Infectious disease	Coxsacchie virus (ICD B34.1) and LOS > =3	All medical and surgical inpatients	
	Pertussis (ICD A37.0, A37.1, A37.8 A37.9) and LOS > =7	LOS > =3, 7 or 14 days appropriate to incubation period	
	Other infections (ICD > =B01, B01.0 and < =B01.9, > = B05. and < =B05.9, B06.0, B06.8, B06.9, B08.4, B15.0 B15.9 > =B26.0 and < =B26.9) and LOS > =14		
Pressure ulcer	Pressure ulcer (ICD L89)	All medical and surgical inpatients	All diagnosis of hemiplegia, paraplegia, paralysis; cerebral palsy; spina bifida. (ICD > =G80 and < =G84; > = Q05. and < =Q05.9 or > =Q07. and < =Q07.03)
		LOS > =3 days	
			All diagnoses of paralysis found in look back period
Failure to rescue	Died in hospital	All medical and surgical inpatients	
		Pneumonia, sepsis, arrest/shock/respiratory failure, LRTI as above and GI bleed and DVT/PE	

*Note.* ICD = international classification of diseases; LOS = length of stay; MDC = major diagnostic category; ACHI = Australian classification of health interventions; AHRQ = agency for healthcare research and quality; DVT/PE = deep vein thrombosis/pulmonary embolus.

Definitions amended from Needleman et al [8], and McCloskey [28].

^**a**^All numerators are based on secondary diagnosis only except surgical wound infection in look forward period.

^b^ICD codes are all ICD-10-AM (Australian Modified).

The aims of our current study were to determine the rates of paediatric nursing-sensitive outcomes in children admitted to the paediatric tertiary hospital in Perth, Western Australia over a 10 year period, and determine the potential clinical utility of measuring nursing-sensitive outcomes in paediatric hospitals using linked hospital data. In addition, we were interested in determining factors associated with children experiencing any of the adverse events.

## Method

### Study design and patients

A retrospective cohort study included all hospitalisation records for Western Australian (WA) children (≤ 18 years), who were admitted to the tertiary paediatric hospital in Perth (WA) for at least one night, during the 10 year period from July 1999 to June 2009. Each child’s hospital record was recorded electronically and was linked to all previous and subsequent electronic hospitalisation records at any public or private hospital in WA (back to 1989 and forward until March 2010) and to the Australian census data, by the Western Australian Data Linkage Branch (WADLB) [29].

Our earlier work used the linked data to develop algorithms for 13 paediatric nursing-sensitive outcomes [21,30]. These algorithms were used to evaluate numerators and denominators for the following outcomes: hospital acquired lower respiratory tract infection other than pneumonia (LRTI); gastrointestinal (GI) infection; pneumonia; sepsis; cardiac/respiratory arrest, shock or respiratory failure; central nervous system (CNS) complication; central venous line (CVL) infection; infectious disease; pressure ulcer; failure to rescue (FTR); surgical wound infection; physiologic/metabolic derangement; and postoperative cardiopulmonary complications. Table 1 provides the criteria for individual paediatric nursing-sensitive outcomes and these algorithms were applied to identify the nursing-sensitive outcomes in this study.

The WA Hospital Morbidity Data (HMD) provided abstracts of demographic and clinical information on all hospital separations from every hospital within WA [31]. Up to 22 diagnoses and 12 procedures are coded for each hospitalisation. Diagnoses were coded using the International Classification of Diseases, Version 10, Australian Modification (ICD-10-AM) [32] and procedures were coded according to the Australian Classification of Health Interventions (ACHI) [33]. Variables for major diagnostic categories (MDC) and Australian Refined Diagnostic Related Groups (AR-DRG) were also provided. The data also included the date, time and ward of every patient’s ward movement within the tertiary hospital. The information from the Australian Census data linked to each HMD record included the accessibility and remoteness index (ARIA+) [34] and the socioeconomic status associated with the residential area of the patient (SEIFA) [35].

The quality of data linkage has been assessed by sampling and the estimated percentage of invalid links (false positives) and missed links (false negatives) was 0.11% [36]. Validation studies for the quality of data in administrative health databases have reported the following percentages: <1% missing demographic information; 87-90% primary diagnoses recorded accurately; > 90% procedures recorded accurately and the accuracy of records of secondary diagnoses varies [36,37]. Data were received in anonymised files and then merged and analysed in SPSS for Windows (Version 19.0.0.1; 2010 IBM SPSS Chicago, Il, USA). Logical checks, such as checking for duplicate records, that hospital separations occurred after hospital admissions, and that each child’s sex, date of birth and ethnicity were the same across databases, were undertaken by the principal researcher during data clean-up. Seventy five records were deleted as they were for adults.

Month and year of birth were provided for each child. Age on admission was calculated and categorised into groups that describe growth, cognitive and psychosocial development (1–28 days = neonate, 29–365 days = infant, > 1-3 years = toddler, > 3-6 years = pre-schooler, > 6-13 years = school-age, > 13- ≤ 18 years = adolescent) [38]. The risk pool for surgical wound infections, postoperative cardiopulmonary complications and physiologic/metabolic derangement was surgical patients only. This subset contained patients classified as 'surgical’ based on AR-DRG codes supplied by WADLB. The remainder of the cohort was classified as 'non-surgical’ and contained children coded as 'medical’ or 'other’. The number of times each patient moved wards during a hospital admission was calculated.

To determine any associations with socioeconomic status, the Index of Relative Socio-Economic Disadvantage (IRSD) was chosen from the Australian Census data as it best represents residential areas of low income, high unemployment and low levels of education [35]. A lower score indicates the family resides where many households in the area were disadvantaged and a higher score indicates the family resides in an area with the least number of households who were disadvantaged. The geographic measure of remoteness (ARIA+) based on accessibility to service centres along road networks was also used. IRSD and ARIA + are constructed from census data collected every five years, therefore scores from the most recent census preceding the hospital admission were used. The IRSD and ARIA + indices are based on geographic levels with the census Collection District (CD) being the smallest spatial unit in the classification. As the IRSD reflect the area in which the family live and not the individual family, CD is most likely to reflect the actual socioeconomic status of the family. Where a CD was not available for an individual, the index for the Statistical Local Area (SLA) was assigned, which is the next smallest spatial unit in the classification [39]. Once assigned, IRSD and ARIA + were categorised into dichotomous variables; least disadvantaged and most disadvantaged, and city and regional or remote WA resident.

Linked data was used to identify the nursing-sensitive outcomes as described in the background. However, when calculating proportions and rates of the outcomes each hospital separation record was assumed to be an independent event; therefore calculations were based on hospital separations or records rather than patients, as in other studies [10,14,15,40].

### Statistical methods

Rates per 10,000 patient days and number per 1,000 hospital separations were calculated for each of the 13 paediatric nursing-sensitive outcomes. The risk pool for surgical wound infection, physiologic/metabolic derangement and postoperative cardiopulmonary complications included surgical records only whereas the risk pool for the other outcomes was medical and surgical records.

Differences between characteristics of those that did and did not acquire a nursing-sensitive outcome were analysed using Chi-square tests for categorical data and independent *t* - tests for continuous data. Poisson regression models were used to determine whether there was a significant association between each nursing-sensitive outcome and each of the potential risk factors: age, sex, type of admission, case mix, number of ward movements, season, year of admission, IRSD and ARIA+. Risk factors which had a statistically significant relationship with one or more nursing-sensitive outcomes were subsequently included in multiple regression models.

For each nursing-sensitive outcome, a Poisson multiple regression model was fitted to estimate the rate ratios (RR) and 95% confidence intervals (CI) for age, sex, admission type, case mix, season, year of admission, IRSD and ARIA+. Except for the nursing-sensitive outcome, surgical wound infection, all Poisson regression models included length of stay (LOS) as an offset variable. As the date of surgery was not supplied in the HMD we were unable to calculate a length of stay post-surgery for surgical wound infection. Significance was set at p < .05.

### Ethical considerations

The project received approval from the Human Research Ethics Committees of the study hospital and the WA Department of Health. Waiver of consent was granted as the study adhered to the National Health and Medical Research Council’s National Statement on Ethical Conduct in Human Research. Permission to use the data for research was granted by the data custodian for each database. Data was de-identified and all personal identifiers were removed before being provided to the researchers. Confidentiality statements were signed by members of the research team. Data files were encrypted when transported and kept securely on a password protected server which was not accessible via the internet consistent with the Practice Code for the use of Personal Health Information [41]. Destruction of the data will adhere to instructions provided in the Australian Government Information Security Manual [42].

## Results

### Patient characteristics

Of 129,719 hospital separations for 79,016 children, 79% were for emergency admissions and 83% were for medical admissions. The number of hospital separations each year ranged from 12,025 in 2003–04 to 14,476 in 2000–01 with a mean number of 12,972/year. The leading major diagnostic category was respiratory disorders, which accounted for 16% of records. Once admitted, 80% of children remained on the same ward throughout their hospital stay. The median LOS (excluding patients who remained less than 1 night) was 2 days, inter-quartile range (IQR) = 1-4 days.

A total of 1,919 (1.5%) hospital separations recorded one or more nursing-sensitive outcomes over the 10 year period. They were experienced by 1,740 children and represented 2,037 occurrences of nursing-sensitive outcomes. Of the separation records that had a nursing-sensitive outcome, 77% were for emergency admissions, 67% were for medical patients and 18% were for the major diagnostic category of gastrointestinal disorder. Once admitted, 62% remained on the same ward throughout their hospital stay and 20% had one within hospital transfer. The median LOS was 6 days and IQR = 4-11 days. There were statistically significant differences between the proportions with each characteristic of those with and without a nursing-sensitive outcome as shown in Table 2.

**Table 2:** Characteristics of hospital separation records with and without a nursing-sensitive outcome (NSO) during 10 years (1999–2009)

	**Separations without any NSO**		**Separations with any NSO**		***p *value**^**a**^
	**(n = 127800)**		**(n = 1919)**		
	***n***	**%**	***n***	**%**	
Age group					< .001
1-28 days (neonate)	5568	4.4	82	4.3	
> 28 days-1 year (infant)	21601	16.9	513	26.7	
> 1-3 years (toddler)	23422	18.3	402	21.0	
> 3-6 years (pre-school)	14620	11.4	166	8.7	
> 6-13 years (school age)	41487	32.5	501	26.1	
> 13- ≤ 18 years (adolescent)	21102	16.5	255	13.3	
Sex					.037
Male	72792	57.0	1139	59.4	
Female	55008	43.0	780	40.7	
Socioeconomic index (IRSD)^b^					< .001
Least disadvantaged	76584	61.1	1031	56.5	
Most disadvantaged	46793	37.9	794	43.5	
Residence (ARIA+)^c^					< .001
Major city	100147	81.2	1365	74.8	
Regional or remote	23189	18.8	460	25.2	
Admission type					.006
Emergency	101229	78.2	1470	76.6	
Elective	26571	20.8	449	23.4	
Case mix					< .001
Non-surgical	106841	83.6	1286	67.0	
Surgical	20959	16.4	633	33.0	
Major Diagnostic Category^c^					< .001
Respiratory	21216	16.6	125	6.5	
Gastrointestinal	16256	12.7	337	17.6	
Musculoskeletal	15358	12.0	204	10.6	
Ear, nose, throat & mouth	13207	10.3	119	6.2	
Neurological	10054	7.9	217	11.3	
Season					< .001
Autumn	31122	24.4	444	23.1	
Winter	34278	26.8	575	30.0	
Spring	33955	26.6	526	27.4	
Summer	28445	22.3	374	19.5	
	**Mean (SD)**		**Mean (SD)**		
Length of stay (LOS)	4.09 (10.62)		11.34 (23.48)		< .001
Ward movements	0.26 (0.64)		0.67 (1.20)		.002

Note: Test statistic is Pearson Chi-square for categorical data and *t*-statistic for continuous data.

^a^ Two sided significance reported.

^b^ Data missing therefore does not equal total.

^c^ Only Major Diagnostic Categories with most cases listed.

### Rates of nursing-sensitive outcomes

There were 113 separation records of the total 129,719 in this study that had more than one nursing-sensitive outcome recorded (0.09%) and there were separation records for 179 children that recorded a nursing-sensitive outcome on more than one separation (0.14%). Table 3 displays the crude (unadjusted) rate of each paediatric nursing-sensitive outcome per 10,000 patient days and the proportion per 1,000 hospital separations. The rate of physiologic and metabolic derangement was greatest at 15.15/10,000 patient days. This was followed by the postoperative cardiopulmonary complications rate of 11.12/10,000 patient days and LRTI and gastrointestinal infections rates of 10.71 and 10.70/10,000 patient days respectively. As proportions of hospital separations, GI infection was 14.01/1,000, surgical wound infection was 10.42/1,000, LRTI was 9.24/1,000 and physiologic and metabolic derangement was 9.07/1,000. Pressure ulcer had the lowest rate at 0.61/10,000 patient days and 0.51/1,000 hospital separations.

**Table 3:** Unadjusted rates for nursing-sensitive outcomes

	**No. of events**	**Total hospital separations**	**No./1000 hospital separations**	**Total patient days**	**Rate/10000 patient days**
LRTI	395	42732	9.24	368835	10.71
GI infection	342	24415	14.01	319683	10.70
Pneumonia	337	102877	3.28	436271	7.72
Surgical wound infection^a^	225	21592	10.42	^___b^	^___b^
Physiologic/metabolic derangement^a^	163	17980	9.07	107594	15.15
Sepsis	136	41807	3.25	373011	3.65
Postoperative cardiopulmonary complications^a^	124	15322	8.09	111468	11.12
Arrest/shock/respiratory failure	94	106369	0.88	447886	2.10
CNS complication	92	87926	1.05	318889	2.89
CVL infection	58	74041	0.78	465663	1.25
Infectious disease	29	52396	0.55	440153	0.66
Pressure ulcer	25	49452	0.51	412555	0.61
Failure to rescue	17	22081	0.77	213316	0.80

*Note.* LRTI = lower respiratory tract infection other than pneumonia; GI = gastrointestinal tract; CNS = central nervous system; CVL = central venous line.

^a^ Risk pool was surgical patients only.

^b^ Result not applicable.

### Risk factors for nursing-sensitive outcomes

Table 4 presents the association between patient sociodemographic characteristics and the risk for each paediatric nursing-sensitive outcome. The risk of developing a LRTI was greater with each decreasing age group. Compared to adolescents, the risk for infants was nearly 8 times greater (RR = 7.74, 95% CI 5.11, 11.75). Winter and spring were also associated with greater risk of developing a LRTI compared to summer (RR = 2.02, 95% CI 1.51, 2.72; and RR = 1.54, 95% CI 1.13, 2.10) respectively.

**Table 4:** Association between sociodemographic characteristics of children and risk of paediatric nursing-sensitive outcomes

	**LRTI**	**Pneumonia**	**GI infect**	**Sepsis**	**Arrest/shock/resp failure**	**CNS complication**	**CVL infect**
No. of events	395	337	342	136	94	92	58
No. in risk pool	42732	102877	24415	41807	106369	87926	74041
	**Rate ratio (95% CI)**	**Rate ratio (95% CI)**	**Rate ratio (95% CI)**	**Rate ratio (95% CI)**	**Rate ratio (95% CI)**	**Rate ratio (95% CI)**	**Rate ratio (95% CI)**
Age							
1-28 days (neonate)	1.52	0.46	0.67	**0.26**	1.11	___^a^	1.02
	(0.84, 2.75)	(0.21, 1.00)	(0.29, 1.58)	**(0.08, 0.90)**	(0.37, 3.36)		(0.35, 3.00)
29-365 days (infant)	**7.74**	**3.51**	**6.56**	**1.95**	**2.36**	**0.29**	0.57
	**(5.11, 11.75)**	**(2.34, 5.25)**	**(4.17, 10.32)**	**(1.07, 3.57)**	**(1.03, 5.37)**	**(0.09, 0.93)**	(0.20, 1.64)
> 1–3 years (toddler)	**7.40**	**4.19**	**9.89**	**3.62**	**5.48**	**0.31**	1.95
	**(4.79, 11.45)**	**(2.78, 6.30)**	**(6.24, 15.69)**	**(1.97, 6.64)**	**(2.57, 11.66)**	**(0.15, 0.65)**	(0.85, 4.46)
> 3–6 years (preschooler)	**5.19**	**2.38**	**3.95**	**2.73**	**2.63**	**0.29**	0.26
	**(3.17, 8.48)**	**(1.47, 3.87)**	**(2.23, 6.98)**	**(1.34, 5.59)**	**(1.04, 6.65)**	**(0.12, 0.75)**	(0.03, 1.97)
> 6-13 years (school-age)	**2.75**	**2.18**	**2.58**	**2.20**	1.92	0.77	2.01
	**(1.76, 4.29)**	**(1.47, 3.24)**	**(1.57, 4.23)**	**(1.23, 3.94)**	(0.87, 4.21)	(0.48, 1.24)	(0.99, 4.04)
> 13- ≤ 18 years (adolescent)	1	1	1	1	1	1	1
Sex							
Male	1.11	1.18	**1.35**	1.08	1.51	1.11	1.22
	(0.90, 1.36)	(0.94, 1.47)	**(1.08, 1.70)**	(0.76, 1.54)	(0.97, 2.34)	(0.73, 1.69)	(0.72, 2.05)
Female	1	1	1	1	1	1	1
Socioeconomic status (IRSD)							
Least disadvantaged	1.20	1.01	0.86	0.99	1.33	1.16	0.99
	(0.98, 1.47)	(0.81, 1.27)	(0.69, 1.08)	(0.70, 1.41)	(0.87, 2.02)	(0.75, 1.78)	(0.59, 1.68)
Most disadvantaged	1	1	1	1	1	1	1
Residence (ARIA+)							
Major city	0.72	0.88	0.96	1.34	0.70	0.78	0.74
	(0.56, 1.00)	(0.68, 1.13)	(0.75, 1.24)	(0.93, 1.94)	(0.41, 1.20)	(0.48, 1.28)	(0.59, 1.68)
Regional or remote	1	1	1	1	1	1	1
Case mix							
Medical	3.01	**0.70**	**3.40**	0.87	**2.08**	**3.69**	0.99
	(2.18, 4.15)	**(0.55, 0.88)**	**(2.26, 5.11)**	(0.58, 1.31)	**(1.11, 3.88)**	**(1.76, 7.72)**	(0.56, 1.76)
Surgical	1	1	1	1	1	1	1
Admission type							
Emergency	1.21	**0.36**	1.00	1.23	0.98	0.78	**0.23**
	(0.91, 1.62)	**(0.29, 0.46)**	(0.73, 1.37)	(0.77, 1.97)	(0.56, 1.73)	(0.48, 1.28)	**(0.14, 0.40)**
Elective	1	1	1	1	1	1	1
Season							
Autumn	1.00	1.28	1.28	0.87	0.81	0.88	1.68
	(0.94, 1.41)	(0.90, 1.82)	(0.90, 1.78)	(0.53, 1.45)	(0.41, 1.63)	(0.48, 1.62)	(0.81, 3.47)
Winter	**2.02**	**1.68**	1.27	0.74	1.64	1.02	0.59
	**(1.51, 2.72)**	**(1.21, 2.34)**	(0.91, 1.77)	(0.45, 1.23)	(0.91, 2.96)	(0.56, 1.84)	(0.24, 1.46)
Spring	**1.54**	**1.58**	1.27	1.07	1.37	1.05	1.53
	**(1.13, 2.10)**	**(1.13, 2.21)**	(0.90, 1.78)	(0.67, 1.73)	(0.74, 2.55)	(0.58, 1.92)	(0.72, 3.25)
Summer	1	1	1	1	1	1	1

*Note.* Multivariable Poisson generalised linear regression analyses, length of stay as log offset and adjusted for year of admission. LRTI = lower respiratory tract infection other than pneumonia; GI = gastrointestinal tract; CNS = central nervous system; CVL = central venous line; IRSD = index of relative socio-economic disadvantage; ARIA + =geographic measure of remoteness. Results in bold indicate statistical significance at p < .05. Pressure ulcer, infectious diseases and failure to rescue not included in table as no statistically significant risks on multivariate analysis.

^a^ Neonates not included in risk pool.

Similarly, the risk of developing pneumonia was greater in younger children, with toddlers at greatest risk. The risk of developing pneumonia was 4 times greater in toddlers than adolescents (RR = 4.19, 95% CI 2.78, 6.30). Compared to summer, there was greater risk in winter (RR = 1.68, 95% CI 1.21, 2.34) and spring (RR = 1.58, 95% CI 1.13, 2.21). Medical patients and emergency admissions had lower risks than surgical and elective admissions of developing pneumonia (RR = 0.70 95% CI 0.55, 0.88 and RR = 0.36, 95% CI 0.29, 0.46) respectively.

The risk of developing GI infection was nearly 10 times greater in toddlers than adolescents (RR = 9.89, 95% CI 6.24, 10.32). The risk was also greater in each age group except neonates. Boys and medical patients were at greater risk (RR = 1.35, 95% CI 1.08, 1.70 and RR = 3.40, 95% CI 2.26, 5.11).

Age was the only factor that was significantly associated with a risk of sepsis. Compared to adolescents, neonates were at a lower risk of developing sepsis (RR = 0.26, 95% CI 0.08, 0.90) and the risks were greatest in toddlers (RR = 3.62, 95% CI 1.97, 6.64). The risk of arrest, shock or respiratory failure, was nearly five and a half times greater in toddlers than adolescents (RR = 5.48, 95% CI 2.57, 11.66). There was a greater risk in each age group except for neonates where there was no association found. Compared to surgical patients, medical patients were twice as likely to arrest, develop shock or respiratory failure (RR = 2.08, 95% CI 1.11, 3.88).

Compared to adolescents, there was a lower risk in occurrence of CNS complications in infants, toddlers and pre-schoolers by approximately one third in each category. The risk in medical patients was 3.69 times greater than surgical patients (95% CI 1.76, 7.72). The only factor to be associated with CVL infections was admission type and the risk in emergency admissions was decreased by 20% (RR = 0.23, 95% CI 0.14, 0.40) compared to children who were elective admissions.

There were no statistically significant risk factors identified for outcomes of failure to rescue, pressure ulcer or infectious diseases. Although significant in one or more univariate models, there were no statistically significant associations between IRSD and ARIA + indices and any of the nursing-sensitive outcomes in multiple regression models.

Table 5 presents the RR and 95% CI for three paediatric nursing-sensitive outcomes that used a risk pool restricted to surgical patients. The risk of developing a surgical wound infection was nearly 8 times greater for emergency admissions compared with elective admissions (RR = 7.78, 95% CI 5.10, 11.86) and lower by over 10% in neonates compared with adolescents (RR = 0.87, 95% CI 0.47, 1.62).

**Table 5:** Association between sociodemographic characteristics of children and risk of paediatric nursing-sensitive outcomes in surgical patients

	**Surgical wound infection**^**a**^	**Physiologic/metabolic derangement**	**Postoperative cardiopulmonary complications**
No. of events	225	163	124
No. in risk pool	21592	17980	15322
	**Rate ratio**	**Rate ratio**	**Rate ratio**
	**(95% CI)**	**(95% CI)**	**(95% CI)**
Age category			
1-28 days (neonate)	0.87	**0.12**	0.55
	(0.47, 1.62)	**(0.04, 0.35)**	(0.28, 1.07)
29-365 days (infant)	1.33	**4.04**	1.20
	(0.82, 2.15)	**(2.32, 7.05)**	(0.64, 2.25)
> 1–3 years (toddler)	0.96	**2.45**	**1.81**
	(0.57, 1.60)	**(1.22, 4.91)**	**(1.01, 3.26)**
> 3–6 years (preschooler)	0.98	1.29	0.47
	(0.57, 1.68)	(0.55, 3.02)	(0.18, 1.23)
> 6-13 years (school-age)	0.84	**1.95**	0.94
	(0.58, 1.23)	**(1.11, 3.43)**	(0.57, 1.55)
> 13- ≤ 18 years (adolescent)	1	1	1
Sex			
Male	0.77	**1.65**	1.21
	(0.59, 1.02)	**(1.17, 2.33)**	(0.83, 1.76)
Female	1	1	1
Socioeconomic status (IRSD)			
Least disadvantaged	1.13	0.90	0.70
	(0.85, 1.50)	(0.65, 1.25)	(0.48, 1.03)
Most disadvantaged	1	1	1
Residence (ARIA+)			
Major city	1.19	1.06	1.00
	(0.87, 1.63)	(0.74, 1.52)	(0.67, 1.50)
Regional or remote	1	1	1
Admission type			
Emergency	**7.78**	**3.46**	**0.38**
	**(5.10, 11.86)**	**(2.32, 5.18)**	**(0.26, 0.55)**
Elective	1	1	1
Season			
Autumn	1.14	0.74	1.02
	(0.77, 1.69)	(0.48, 1.15)	(0.61, 1.73)
Winter	1.09	0.65	1.27
	(0.73, 1.64)	(0.42, 1.03)	(0.77, 2.09)
Spring	1.09	0.90	0.87
	(0.73, 1.64)	(0.58, 1.42)	(0.50, 1.52)
Summer	1	1	1

*Note.* Multivariable Poisson generalised linear regression analyses, length of stay as log offset and adjusted for year of admission. IRSD = index of relative socio-economic disadvantage; ARIA + =geographic measure of remoteness. Results in bold indicate statistical significance at p < .05.

^a^ Length of stay not used as an offset variable.

Compared to adolescents, the risk of developing physiologic and metabolic derangement was 4.04 times greater in infants (95% CI 2.32, 7.05) and it was also lower in toddlers (RR = 2.45, 95% CI 1.22, 4.91) and school-age children (RR = 1.95, 95% CI 1.11, 3.43). The risk was almost 90% lower in neonates (RR = 0.12, 95% CI 0.04, 0.35). The risk was 1.65 times greater in boys (95% CI 1.17, 2.33) and 3.46 times in children who were emergency admissions (95% CI 2.32, 5.18).

The risk of postoperative cardiopulmonary complications was nearly twice as great in toddlers as adolescents (RR = 1.81, 95% CI 1.01, 3.26), but risks in other age groups did not differ significantly. The risk associated with emergency admission was nearly 60% less than that of elective admission (RR = 0.38, 95% CI 0.26, 0.55). Sex, season, IRSD and ARIA + indices were not statistically significantly associated with any of the surgical nursing-sensitive outcomes.

## Discussion

Application of our 13 'paediatric’ nursing-sensitive outcome algorithms [21] identified a limited number of nursing-sensitive outcomes that had more than 100 events over the 10 year period. They were: hospital acquired infections of LRT, GI tract, surgical wounds, sepsis and pneumonia, and physiologic/metabolic derangement and postoperative cardiopulmonary complications. Incidences ranging from 3-14/1,000 hospital separations suggest that they may occur frequently enough to be useful as measures of quality nursing care. The minimum number of events for clinical utility has not been defined to date, however the Canadian Institute for Health Information [43] recommends that a 'cut off’ or a minimum number of events for each outcome be discussed with consideration of its value for a meaningful and feasible analysis. Further consideration regarding how to report information when numbers of events are small is also warranted [43,44].

The low incidence rates for arrest/shock/respiratory failure, CNS complications, CVL infection, infectious disease, pressure ulcer and failure to rescue suggest that either the algorithms are not sensitive enough to accurately identify the number of nursing-sensitive outcomes, that there are insufficient numbers of outcomes recorded, or insufficient numbers of outcomes occurred, suggesting the usefulness of these outcomes as quality indicators in paediatric nursing may be questionable. The rates found in this study for pressure ulcer, FTR and infectious disease are lower than those reported in other studies [40,45-49]. The administrative hospital data used to identify rates of pressure ulcer and FTR in these other studies was not linked when identifying records with nursing-sensitive outcomes of pressure ulcer and FTR which may account for their higher rates; however adjustments were made for comorbidities [40,45-48]. Since the reporting of rates of hospital acquired infectious diseases in an Australian point prevalence study [49], the national immunisation schedule has increased the range of vaccines freely available to Australians. Consequently, the increased level of immunity has decreased the chances of acquiring infectious diseases both in the community and in hospital. Although a 'cut off’ for a minimum number of events has not been stipulated, these studies consistently conclude that there are currently insufficient events in paediatric populations to be clinically useful for measuring quality [40,50].

Five of the seven nursing-sensitive outcomes that could be considered for use, include hospital acquired infections (LRTI, GI infection, pneumonia, sepsis, surgical wound infection). Age was significantly associated with risks of developing these infections except for surgical wound infections. Neonates and infants have immature immune systems and are more prone to infections. However, compared with adolescents, neonates had similar risks of nursing-sensitive infections, but risks were high for infants, and even higher for toddlers. The study hospital accommodates neonates and infants in areas specific to their age, which may increase awareness of their vulnerability and specific needs for infection control precautions. Parents may also be more aware of the vulnerability of young children to infection and do not visit or allow visitors if they have an infection. Infants and neonates are not independently mobile so are confined to cots and their rooms whereas older children are mobile and their movement around ward areas and interaction with other patients and visitors could contribute to their greater risk. In addition, the airway of a child under approximately eight years of age differs anatomically to that of an adolescent or adult. These anatomical differences put neonates, infants, toddlers and pre-schoolers at greater risk of developing LRTI, pneumonia and arrest/shock/respiratory failure as shown in our results. Therefore it is important to consider age when comparing these nursing-sensitive outcomes between agencies or between units. Age was stratified to represent the accepted developmental groups [38] and our results, which report comparisons between groups, are considered to be more useful than comparisons based on annual changes.

Seasonal differences from summer occurred in winter and spring for rates of LRTI and pneumonia. The viruses causing these conditions tend to be more prevalent in the community and increase the chances of infection during these seasons. Accordingly, children’s hospital inpatient numbers increase during winter and spring. Further research would be useful to determine if levels of nurse staffing, such as nurse patient ratio or nursing hours per patient day, alter during the busy seasons and whether there is any association with the nursing-sensitive outcomes. Stratton [50] measured five nursing-sensitive outcomes for each quarter and found a significant association between winter and parent/family complaints. Twigg [15] adjusted for season when seeking associations between nurse staffing and nursing-sensitive outcomes in an adult population based on conceptual grounds but did not report whether there were any associations.

An important reason for measuring the quality of nursing care is to identify areas where the care can be improved by implementing evidence informed practices. Where there is evidence of lowering rates of nursing-sensitive outcomes by initiating the same 'bundle of care’ or changes in nurses’ behaviour related to the bundle of care, consideration can be given to combining results and reporting the rates as a composite indicator. For example, LRTI, GI infection and infectious disease are related to adherence of infection control procedures, a bundle of care, such as hand hygiene, assessment of patient history, patient isolation and family education [51]. Therefore, 'grouping’ the results of these nursing-sensitive outcomes may make implementation of practice changes easier and monitoring more straightforward during quality improvement activities. Similarly, rates of sepsis, surgical wound infection, and CVL infection are related to hand hygiene and patient/family education; consequently, these outcomes could be grouped and reported for surgical patients.

As well as providing evidence informed care that is safe and effective, nurses are increasingly accountable to the public for the expenditure of their care [52,53]. Therefore it is necessary to consider the significant difference in the hospital length of stay (LOS) between children who did and did not develop a nursing-sensitive outcome. It is unclear whether the increased LOS occurred as a result of the nursing-sensitive outcome or whether the children developed the nursing-sensitive outcome as a result of being in hospital longer. There was also a significant difference in number of ward movements. Finally, the increased number of ward movements in those who developed a nursing-sensitive outcome may be a result of the nursing-sensitive outcome rather than the ward movements or 'churn’ contributing to the outcome [54]. Many of the outcomes measured were infectious so children would need to be moved to an area where they could be isolated from non-infected children or to an intensive care area if the child’s airway and respiratory system were compromised. This data was not available.

Using administrative data to measure nursing-sensitive outcomes has both advantages and limitations. Advantages are that the data collection follows national standards and it provides the groundwork for benchmarking and comparisons of rates between the paediatric hospitals in Australia, the data is collected routinely reducing the need to collect information from case records or regular audits which is time consuming and often takes nurses away from providing nursing care [55]. Limitations are that the outcomes measured are not necessarily those that best reflect the quality of nursing care and there may be other patient outcomes that provide this; [30] and the inability to determine whether secondary diagnoses are pre-existing comorbid conditions or complications that occurred during hospitalisation, which increases the likelihood of under or over reporting of outcomes. Under reporting tends to occur when the outcomes have no financial implication for the health service provider [56,57]. The advantages of feasibility, cost saving and having complete longitudinal population data when using administrative health data offsets the limitations and can provide reliable population-based estimates of nursing-sensitive outcomes. These implications of using administrative data have been discussed in our previous work [21].

### Limitations

Firstly, patient’s conditions present on admission are not all routinely recorded in the separation abstract of WA HMD. Therefore, the algorithms to identify the nursing-sensitive outcomes remove some children that are at high risk of developing the outcome. As a consequence, application of these algorithms may not provide a true indication of the quality of nursing care. However, if the purpose of measuring the outcomes is for quality improvement or to provide a benchmark, and the same algorithms are used, rates could be comparable.

Secondly, an illness severity score or comorbidity index has not been used in this study. As risk adjustment must be context specific [58] and no paediatric comorbidity index has been validated, identification of comorbid conditions, not identified in algorithms, that increase the risk of a child developing a nursing-sensitive outcome was not undertaken.

Thirdly, although patient hospital records were linked to identify the nursing-sensitive outcomes, each hospital record with any nursing-sensitive outcomes was considered an individual event. There is a chance that the same nursing-sensitive outcome has been counted twice. However, it could be argued that if a child is readmitted with a nursing-sensitive outcome, it should still be counted as a separate outcome as it could be related to inappropriate discharge planning and suboptimal child and family education which are fundamental nursing roles.

Fourthly, we were only able to consider risk factors that are available in the data collected. There may be other factors associated with the quality of care that influence nursing-sensitive outcomes such as processes of care. Structures that influence care such as levels of nurse staffing and experience and education of nurses need to be considered.

Finally, neither ethnicity nor race was included in any models. Although both have been demonstrated to be associated with greater rates of inpatient safety indicators in studies in adult populations [59], associations with poorer health outcomes in paediatric studies have not been demonstrated [60]. If hospital care is equitable, the incidence of nursing-sensitive outcomes should not vary between racial or ethnic groups and this warrants further research. Unfortunately, indigenous status is not a reliable variable in HMD [61-63] precluding meaningful analysis.

### Implications for policy and practice

There are sufficient numbers of events of some paediatric nursing-sensitive outcomes to make them useful measures of quality of nursing care for quality improvement and to benchmark with other Australian tertiary paediatric hospitals. This information can lead to evidence informed practice as nurses’ share their practices and strive to minimise the numbers of adverse patient events. Future research can focus on comparisons with other paediatric hospitals to understand variability in outcome rates. Comparisons with adult hospitals can be undertaken to determine variability with children’s hospitals.

To assist in the accurate identification of hospital acquired complications, it is recommended that a 'present on admission’ variable be recorded in the separation abstract, as used in California and Canada [64]. Such a variable would eliminate the need for complex algorithms which have the potential of removing children who are at high risk of getting the outcome from the risk pool. Children who are at high risk of getting the outcome are the children we need to be including in the quality improvement exercise.

Risks of developing the nursing-sensitive outcomes investigated tend to decrease as the child gets older. Reporting of nursing-sensitive outcomes should be stratified by developmental age groups to better reflect the areas to target for quality improvement. As well as comorbidities or severity of illness, factors that have not been considered are the levels of nurse staffing and the education levels of nurses. Many studies have shown relationships between rates of nursing-sensitive outcomes and levels of nurse staffing predominantly in adult hospitals [8,9,11-15] with minimal paediatric studies [40,50]. As paediatric nursing-sensitive outcomes are available, further study of their association with levels of nurse staffing can now be undertaken.

In addition, if nursing-sensitive outcomes are to be used for benchmarking between hospitals, adjusting for hospital characteristics such as bed number, teaching status, number of intensive care beds and case mix needs to be considered. Such adjustments were not required for this study as it was carried out in one children’s hospital.

## Conclusion

In conclusion, there are adequate numbers of events and incidence for LRTI, GI infection, pneumonia, surgical wound infection, physiologic/metabolic derangement, sepsis and postoperative cardiopulmonary complications for them to be considered useful nursing-sensitive outcomes to contribute to the measurement of quality paediatric nursing care. When used for quality improvement or to benchmark with other agencies, data needs to be adjusted for age or stratified by age to ensure equitable comparisons. Other characteristics that may alter the risks for children developing a nursing–sensitive outcome and require consideration include sex, admission type, whether the admission type is medical or surgical, and season. Ultimately the goal of implementing evidence informed nursing practice should be targeted to groups most at risk of developing a nursing-sensitive outcome.

## Competing interests

The authors declare that they have no competing interests.

## Authors’ contributions

SW conceived the study, secured funding, carried out the study design, data analyses, drafted the manuscript. AB assisted with data analyses, revised manuscript. YH revised manuscript. JF conceived the study, secured funding, assisted with data analyses, revised manuscript. All authors read and approved the final manuscript.

## Pre-publication history

The pre-publication history for this paper can be accessed here:

http://www.biomedcentral.com/1472-6963/13/396/prepub

## References

[B1] Australian Institute of Health and Welfare Australia’s health 2010 2010 Canberra ACT (Australia): AIHW

[B2] Council of Australian Governments National healthcare agreement 2008 Canberra: COAG 1 33

[B3] Labour force-healthhttp://www.aihw.gov.au/labourforce/health.cfm

[B4] Nursing-sensitive indicators http://www.nursingworld.org/MainMenuCategories/ThePracticeofProfessionalNursing/PatientSafetyQuality/Research-Measurement/The-National-Database/Nursing-Sensitive-Indicators_1.aspx

[B5] Nursing matters fact sheet: nursing sensitive outcome indicators http://www.icn.ch/images/stories/documents/publications/fact_sheets/15c_FS-Nursing_Sensitive_Outcome_Indicators.pdf

[B6] Yang KPA Simms LM Yin JCT Factors influencing nursing-sensitive outcomes in Taiwanese nursing homes Online J Issues Nurs 1999 4 2

[B7] AHRQuality indicators: introductionhttp://www.qualityindicators.ahrq.gov/

[B8] Needleman J Buerhaus PI Potter V Mattke S Stewart M Zelevinsky K Nurse staffing and patient outcomes in hospital 2001 Boston: Harvard School of Public Health i-487

[B9] Aiken LH Clarke SP Sloane DM Lake ET Cheney T Effects of hospital care environment on patient mortality and nurse outcomes J Nurs Adm 2008 38 5 10.1097/01.NNA.0000312773.42352.d7 PMC2586978 18469615

[B10] Needleman J Buerhaus P Pankratz VS Leibson CL Stevens SR Harris M Nurse staffing and inpatient hospital mortality N Engl J Med 2011 364 11 10.1056/NEJMoa1009492 21410372

[B11] Tourangeau AE Tu JV Developing risk-adjusted 30-day hospital mortality rates Res Nurs Health 2003 26 6 10.1002/nur.10110 14689464

[B12] Van den Heede K Sermeus W Diya L Clarke SP Lesaffre E Vleugels A Aiken LH Nurse staffing and patient outcomes in Belgian acute hospitals: cross-sectional analysis of administrative data Int J Nurs Stud 2009 46 7 928 939 10.1016/j.ijnurstu.2008.05.007 18656875 PMC2700208

[B13] McCloskey BA Diers DK Effects of New Zealand’s health reengineering on nursing and patient outcomes Med Care 2005 43 11 10.1097/01.mlr.0000182549.85761.cd 16224308

[B14] Duffield C Diers D O’Brien-Pallas L Aisbett C Roche M King M Aisbett K Nursing staffing, nursing workloa21d, the work environment and patient outcomes Appl Nurs Res 2011 24 244 255 10.1016/j.apnr.2009.12.004 20974086

[B15] Twigg D Duffield C Bremner A Rapley P Finn J The impact of the nursing hours per patient day (NHPPD) staffing method on patient outcomes: a retrospective analysis of patient and staffing data Int J Nurs Stud 2011 48 5 10.1016/j.ijnurstu.2010.07.013 20696429

[B16] Twigg D Duffield C Thompson PL Rapley P The impact of nurses on patient morbidity and mortality: the need for a policy change in response to the nursing shortage Aust Health Rev 2010 34 3 10.1071/AH09655 20797363

[B17] Blegen M Goode C Reed L Nurse staffing and patient outcomes Nurs Res 1998 47 1 10.1097/00006199-199801000-00001 9478183

[B18] Shuldham C Parkin C Firouzi A Roughton M Lau-Walker M The relationship between nurse staffing and patient outcomes: a case study Int J Nurs Stud 2009 46 7 10.1177/002088171004600203 18675419

[B19] Sochalski J Is more better? The relationship between nurse staffing and the quality of nursing care in hospitals Med Care 2004 42 Suppl 2 II-67 II-73 14734944 10.1097/01.mlr.0000109127.76128.aa

[B20] Schwalenstocker E Bisarya H Lau S Adebimpe O Nursing-sensitive indicators for children’s hospital care quality: a white paper prepared by the Pediatric Data Quality Systems (Pedi-QS) Collaborative 2007: US Pediatric Data Quality Systems (Pedi-QS) Collaborative 1 198

[B21] Wilson S Bremner AP Hauck Y Finn J Identifying paediatric nursing-sensitive outcomes in linked administrative health data BMC Health Serv Res 2012 12 209 10.1186/1472-6963-12-209 22818363 PMC3467158

[B22] National Centre for Classification in Health Australian coding standards 2008 6 Sydney: University of Sydney

[B23] Beal AC Co JPT Dougherty D Jorsling T Kam J Perrin J Palmer RH Quality measures for children’s health care Pediatrics 2004 113 1 10.1542/peds.113.1.1 14702502

[B24] Charlson ME Pompei P Ales KL MacKenzie CR A new method of classifying prognostic comorbidity in longitudinal studies: development and validation J Chronic Dis 1987 40 5 10.1016/0021-9681(87)90171-8 3558716

[B25] Elixhauser A Steiner C Harris DR Coffey RM Comorbidity measures for use with administrative data Med Care 1998 36 1 10.1097/00005650-199801000-00001 9431328

[B26] McDonald KM Davies SM Haberland CA Geppert JJ Ku A Romano PS Preliminary assessment of pediatric health care quality and patient safety in the United States using readily available administrative data Pediatrics 2008 122 2 10.1542/peds.2007-2477 18676529

[B27] Pediatric quality indicators technical specifications - version 4.4 2012 http://www.qualityindicators.ahrq.gov/Modules/PDI_TechSpec.aspx

[B28] McCloskey BP The effects of health policy on nursing and patient outcomes in New Zealand [dissertation] 2003 School of Nursing: Yale University

[B29] Data linkage Western Australiahttp://www.datalinkage-wa.org/

[B30] Wilson S Hauck Y Bremner A Finn J Quality nursing care in Australian paediatric hospitals: a Delphi approach to identifying indicators J Clin Nurs 2012 21 11 12 10.1111/j.1365-2702.2011.03739.x 22416966

[B31] Government of Western Australia Department of Health Hospital morbidity data system reference manual 2010 Perth: Government of Western Australia

[B32] National Centre for Classification in Health The international statistical classification of diseases and related health problems, tenth revision, Australian modification (ICD-10-AM) 2008 6 Sydney: University of Sydney

[B33] National Centre for Classification in Health Australian classification of health interventions 2008 6 Sydney: University of Sydney

[B34] About ARIA + (accessibility/remoteness index of Australia) http://www.adelaide.edu.au/apmrc/research/projects/category/about_aria.html24124641

[B35] Adhikari P Socio-economic indexes for areas: introduction, use and future directions Analytical Services 2006 Canberra, ACT (Australia): Australian Bureau of Statistics 1 48

[B36] Holman CDAJ Bass AJ Rouse IL Hobbs MST Population-based linkage of health records in Western Australia: development of a health services research linked database Aust N Z J Public Health 1999 23 5 10.1111/j.1467-842x.1999.tb01297.x 10575763

[B37] Henderson T Shepheard J Sundararajan V Quality of diagnosis and procedure coding in ICD-10 administrative data Med Care 2006 44 11 10.1097/01.mlr.0000228018.48783.34 17063133

[B38] Child developmenthttp://www.cdc.gov/ncbddd/childdevelopment/positiveparenting/index.html

[B39] Australian Bureau of Statistics Socio-economic indexes for areas (SEIFA) - technical paper 2006 2008 Canberra, ACT (Australia): Australian Bureau of Statistics 1 76

[B40] Mark BA Harless DW Berman WF Nurse staffing and adverse events in hospitalized children Policy Polit Nurs Pract 2007 8 2 10.1177/1527154407303499 17652626

[B41] Department of Health Western Australia Practice code for the use of personal health information 2009 Perth WA (Australia): Government of Western Australia 1 18

[B42] Department of Defence Intelligence and Security Australian government information security manual 2011 Canberra, ACT (Australia): Commonwealth of Australia 1 306

[B43] Canadian Institute for Health Information Hospital standardised mortality ratio (HSMR) technical notes Ottawa 2011 Ontario: CIHI 1 17

[B44] Australian Commission on Safety and Quality in Health Care National core, hospital-based outcome indicator specification (2012). version 1.1. Consultation draft 2012 Sydney: ACSQHC 1 97

[B45] Miller MR Zhan C Pediatric patient safety in hospitals: a national picture in 2000 Pediatrics 2004 113 6 10.1542/peds.113.6.1741 15173500

[B46] Sedman A Harris JM II Schulz K Schwalenstocker E Remus D Scanlon M Bahl V Relevance of the agency for healthcare research and quality patient safety indicators for children’s hospitals Pediatrics 2005 115 1 15579669 10.1542/peds.2004-1083

[B47] Kronman MP Hall M Slonim AD Shah SS Charges and lengths of stay attributable to adverse patient-care events using pediatric-specific quality indicators: a multicenter study of freestanding children’s hospitals Pediatrics 2008 121 6 10.1542/peds.2007-2831 18519468

[B48] Scanlon MC Harris JM II Levy F Sedman A Evaluation of the agency for healthcare research and quality pediatric quality indicators Pediatrics 2008 121 6 10.1542/peds.2007-3247 18474532

[B49] Burgner D Dalton D Hanlon M Wong M Kakakios A Isaacs D Repeated prevalence surveys of paediatric hospital-acquired infection J Hosp Infect 1996 34 3 10.1016/s0195-6701(96)90062-6 8923270

[B50] Stratton KM Pediatric nurse staffing and quality of care in the hospital setting J Nurs Care Qual 2008 23 2 10.1097/01.NCQ.0000313758.33654.49 18344775

[B51] Cruickshank M Ferguson J Cruickshank M, Ferguson J Reducing harm to patients from health care associated infection: the role of surveillance Australian Commission on Safety and Quality in Health Care 2008 Sydney: 374

[B52] Alexander GR Nursing sensitive databases: their existence, challenges, and importance Med Care Res Rev 2007 64 2 suppl 44S 63S 10.1177/1077558707299244 17406011

[B53] Health reform implementation taskforcehttp://www.health.wa.gov.au/hrit/docs/publications/clinicalframework.pdf

[B54] Duffield C Diers D Aisbett C Roche M Churn: patient turnover and case mix Nurs Econ 2009 27 3 19558079

[B55] National Health Service (UK) From the impact of nursing on patient clinical outcomes: developing quality indicators to improve care [internet] 2005 Glasgow: NHS

[B56] Lawthers AG McCarthy EP Davis RB Peterson LE Palmer RH Iezzoni LI Identification of in-hospital complications from claims data: is it valid? Med Care 2000 38 8 10.1097/00005650-200008000-00003 10929991

[B57] Needleman J Buerhaus P Mattke S Stewart M Zelevinsky K Nurse-staffing levels and the quality of care in hospitals N Engl J Med 2002 346 22 10.1056/NEJMsa012247 12037152

[B58] Iezzoni LI Risk adjustment for measuring health care outcomes 2003 3 Washington DC: AcademyHealth

[B59] Coffey RM Andrews RM Moy E Racial, ethnic, and socioeconomic disparities in estimates of AHRQ patient safety indicators Med Care 2005 43 Suppl 3 I-48 I-57 15746591 10.1097/00005650-200503001-00008

[B60] Jutte DP Roos LL Brownell MD Administrative record linkage as a tool for public health research Annu Rev Public Health 2011 32 92 108 10.1146/annurev-publhealth-031210-100700 21219160

[B61] Fremantle E Zurynski YA Mahajan D D’Antoine H Elliott EJ Indigenous child health: urgent need for improved data to underpin better health outcomes Med J Aust 2008 188 10 10.5694/j.1326-5377.2008.tb01797.x 18484933

[B62] Australian Institute of Health and Welfare Indigenous identification in hospital separations data–quality report 2010 Canberra, ACT (Australia): AIHW 1 68

[B63] Australian Institute of Health and Welfare Indigenous identification in hospital separations data. Volume 25 2005 Canberra, ACT (Australia): AIHW 1 135

[B64] Hughes JS Averill RF Goldfield NI Gay JC Muldoon J McCullough E Xiang J Identifying potentially preventable complications using a present on admission indicator Health Care Financ Rev 2006 27 3 PMC4194950 17290649

